# The effects of a music intervention on the autonomic nervous system during recovery from strenuous exercise

**DOI:** 10.1111/anec.13096

**Published:** 2023-11-20

**Authors:** Mingyang Niu, Ruixue Zhao, Jiameng Wang

**Affiliations:** ^1^ Yongin University, Graduate School Yongin‐si Gyeonggi‐do Korea; ^2^ Lu Xun Art College Yan'an University Yan'an Shanxi China; ^3^ Faculty of Physical Education Yan'an University Yan'an Shanxi China

**Keywords:** heart rate recovery, heart rate variability, music intervention, myocardial infarction, sudden cardiac death

## Abstract

**Objective:**

To investigate the effect of music on heart rate recovery (HRR) and heart rate variability (HRV) after intense exertion.

**Methods:**

Five hundred male students enrolled at Yongin University, Korea, underwent a cycling test to assess aerobic capacity; 180 students with equal scores were selected for a music intervention, which was conducted after vigorous exercise. The 180 participants were randomized into three music groups and a control group; the participants in each music group listened to music at three different tempos: slow (*lento*) (*n* = 45), moderate (*moderato*) (*n* = 45), and fast (*allegretto*) (*n* = 45). The control group did not listen to music (*n* = 45). After the test, data on cardiac recovery and HRV were gathered and modeled.

**Results:**

The results revealed no significant variation in HRR and HRV indexes between the four cohorts (*p* > .05), and no significant differences were observed in the anaerobic power cycling indexes during strenuous exercise (*p* > .05). The music intervention had a significant impact on HR, low‐frequency power (LF), high‐frequency power (HF), normalized LF (LF_norm_), normalized HF (HF_norm_), and the LF/HF ratio during recovery (*p* < .05).

**Conclusion:**

After rigorous activity, listening to *allegretto* music improved HRR and restored HRV equilibrium, which is critical to preventing and minimizing arrhythmias and sudden cardiac death.

## Graphical abstract

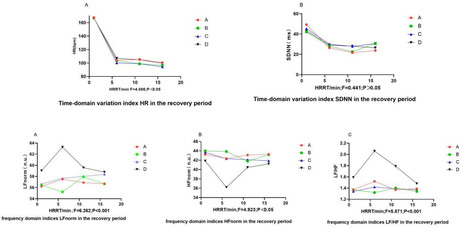

## 1 INTRODUCTION

According to a 2018 poll by the American College of Sports Medicine (ACSM) (American College of Sports Medicine, 2018), the yearly death rate for African American elite athletes was 1 in 40,000 in men and 1 in 50,000 in women (Harmon et al., 2014; Maron et al., 2014; Van Camp et al., 1995). From 2000 to 2015, the rate of premature fatalities due to fitness‐related activities in South Korea was 1 in 50,000, with 32 men and 1 woman among the 33 total casualties (Shun‐Zhe et al., 2015). The proportion of sudden deaths during exercise in college students rose steadily from 2007 to 2016, with men displaying a greater risk than women. Cardiovascular incidents caused by vigorous exercise are also a major concern in other countries, and efficient therapeutic techniques are crucial to mitigating the risk of fatal injury (Lv & Zheng, 2020).

Cardiovascular episodes evoked by strenuous exertion can be categorized as sudden cardiac death (SCD) or acute myocardial infarction (AMI) (Maron et al., 2009; Moore et al., 2016); the occurrence of these episodes is directly correlated with the condition of the body before physical exercise, the intensity of the exercise session, the duration of the exertion, and the recovery process after exercise. Rapid, intense exercise can dramatically increase the risk of SCD and AMI (Willich et al., 1993; Wu, 2019). From a physiological metabolic perspective, strenuous activity is a collective term for short‐duration, vigorous, energy‐supplying exercise that is enabled by two metabolic systems: the adenosine triphosphate–phosphocreatine (ATP–PC) system and anaerobic glycolysis (Atanasovska et al., 2018; Wu & Feng, 1997). Strenuous activity triggers a mild increase in potassium ion concentration in the lungs, and during the rehabilitation period, temporary mild decreases in body potassium concentration occur; this impairs cardiac repolarization and causes rapid changes in the cardiac sympathetic–vagal balance. During the period between exertion and recovery, rapid alterations of autonomic tone and potassium ion concentrations, along with the intrinsic electrophysiological properties of myocardial ion channels, may cause arrhythmias or SCD in susceptible individuals (Huang et al., 2015).

Heart rate variability (HRV) describes the variation in the time between heartbeats and is monitored by the length of two consecutive RR intervals. Heart rate recovery (HRR) and HRV are noninvasive measures that primarily represent autonomic nerve fiber control; parasympathetic nerves have been associated with the rate of recovery of vital signs after cardiac events (Fan et al., 2019; Li et al., 2020; Ma et al., 2008; Wang et al., 2020). Music has been shown to alter HRR and HRV by modulating overactive hormonal indicators (Thomson & Garvie, 1981), approximately doubling excitability at the onset of endurance training and leading to increased metabolism, increased stroke volume, and vasoconstriction; by contrast, parasympathetic nerves play a different role if they are in a depressed state (Goldberger et al., 2001). To observe the effect of music on HRR and HRV and determine which music tempo has the greatest effect on the recovery and homeostatic regulation of HRR and HRV after strenuous exercise, we investigated cardiovascular‐related events in young adults with a high fitness level after strenuous physical exercise through a music intervention.

## 2 METHODS

### 2.1 General information

A total of 500 male college students attending Yongin University, South Korea, on September 1, 2019, were randomly selected as the study population.

The inclusion criteria were as follows: (1) the students were registered and enrolled in the university's student administration center as of September 1, 2019; (2) the students knowingly consented and voluntarily participated in this cohort study.

The exclusion criteria were cardiovascular ailments or other illnesses that would prevent the student from undergoing strenuous, high‐intensity power cycling trials.

### 2.2 Methods and observation indexes

#### 2.2.1 Grouping methodology

The 500 respondents underwent anaerobic power cycling tests; on the basis of their scores, 180 students with similar scores were selected as participants and randomly divided into four subgroups: group A, the slow (*lento*) group (45); group B, the medium (*moderato*) group (45); group C, the fast (*allegretto*) group (45); and group D, the control group (45). One‐way analysis of variance (ANOVA) was conducted, and the subgroups were adjusted to ensure that there were no significant differences in anaerobic power cycling scores between groups.

#### 2.2.2 Fundamental profile collection

General demographic information (age, gender, height, weight, and body mass index [BMI]) was collected from the 180 participants using a questionnaire developed for this study.

#### 2.2.3 Power cycling test

The power cycling test was conducted by two professionals independently; the relevant test methods and precautions were explained to each participant before the test. Using a power bicycle (Lode, Netherlands), the participants were allowed to warm up by pedaling for 2 min. The exercise load was based on the participant's body mass (0.083 kp/kg) (Arena et al., 2010), and each participant performed high‐intensity anaerobic exercise for 30 s at their maximum heart rate. The participants wore a heart rate chest strap (Polar, Finland) to ensure that their heart rate intensity was greater than 85% of their maximum heart rate for the entire cycling test and to record the test results.

#### 2.2.4 HRR and HRV testing

The HRR and HRV testing was completed independently by two professionals, and the relevant test methods and precautions were explained to each participant before the test. The HRV measurement system Ubpuse T1 (Biospace, Korea) was used to measure the HRV index 0–1 min before the warm‐up. During the recovery period (0–16 min after the cycling test), the participants in all four groups listened to piano songs on a headset (Sony WH‐1000XM3, Japan) at four different speeds according to their group assignment, as shown in Table 2. A timer was started immediately after the cycling test ended, and each participant's HRV index was measured 0–1, 5–6, 10–11, and 15–16 min after exercise (see Table 1).

**TABLE 1:** Heart rate variability index parameters.

Heart rate variability indicator	Definition	Parameter meaning
HR (beats/min)	Average heart rate	Reflects the mean of the RR interval
SDNN (ms)	Standard deviation of all normal adjacent RR intervals	Simple measure of autonomic activity
LF (ms^2^)	Low‐frequency power	Reflects sympathetic nerve activity
HF (ms^2^)	High‐frequency power	Reflects the activity of parasympathetic nerves
LF_norm_ (n.u.)	LF/(LF + HF) × 100	Directly reflects changes in sympathetic regulation
HF_norm_ (n.u.)	HF/(LF + HF) × 100	Directly reflects changes in parasympathetic regulation
LF/HF	Low‐frequency power/high‐frequency power	Sympathetic and parasympathetic balance

#### 2.2.5 Track name and beat test

One researcher completed the track name and beat test and, according to the measurement specifications, used an electronic metronome (Seiko SP70, Japan) to test the average tempo of the piano tracks and recorded the results (see Table 2).

**TABLE 2:** Track information.

Track name	Average speed (beats/min)	Speed mark	Duration (min)
Chopin Etude, Opus 25 No. 7	52	Slow (*lento*)	5.33
Schumann: Kinderszenen, Dreaming, Opus 15 No. 7	88	Medium (*moderato*)	3.17
Mozart Sonata in C Major, Movement III, K. 330	115	Fast (*allegretto*)	5.00

#### 2.2.6 Participant consent and experimental setup

At the beginning of the experiment, all participants were informed of the precautions, specific test time, and test plan. To minimize the influence of external factors on the experimental results, the environment of this experiment was kept quiet. The temperature in the laboratory was maintained at 23°C, and the relative humidity was maintained at 65%; the test period was fixed, and the time at which the experiment was conducted was 15:00–17:00. Six participants were tested per day. The participants were prohibited from listening to other music, participating in vigorous exercise, eating, and drinking caffeinated beverages for 3 h before the experiment, they were asked to abstain from drinking alcohol for 48 h before the experiment. During the 0–16‐min recovery period, the track was set to a single‐track loop mode and played continuously, and the volume of the track was set to 60 decibels. The relevant experimental data were measured, recorded, and saved (Dimopoulos et al., 2011; Riganello et al., 2019; Tucker et al., 2014).

### 2.3 Data entry and statistical analysis methods

The data were entered and checked in two ways. For missing data, if the missing data accounted for less than 1/3 of the total data, the average of the available data was used to replace the missing data; if the missing data accounted for over 1/3 of the total data, they were omitted. The input data were sorted in Excel, and SPSS 22.0 was used for data analysis. The general data were described by frequencies and percentages, and the quantitative data with normal distributions were represented by the representation. Repeated one‐way ANOVA was performed to assess the differences in the anaerobic power cycling test indicators (HRR and HRV) between groups before and during high‐intensity exercise. Repeated two‐way ANOVA was used to assess the differences in HRR and HRV between groups during the recovery period. The hypothesis testing criterion was set to *p* < .05 to determine parameters with significant differences.

## 3 RESULTS

### 3.1 Basic information

The basic information about the participants is presented in Table 3.

**TABLE 3:** Physical morphological characteristics of 173 participants ($\bar{x}\pm s$).

Object (*N*)	Age (years)	Height (cm)	Weight (kg)	BMI (kg/m^2^)
Group A (44)	22.89 ± 3.91	173.67 ± 6.74	74.18 ± 13.62	24.15 ± 3.31
Group B (44)	23.64 ± 4.14	173.94 ± 5.65	72.69 ± 11.73	23.91 ± 3.24
Group C (41)	23.44 ± 4.20	173.73 ± 6.76	74.41 ± 13.17	24.60 ± 3.45
Group D (44)	22.59 ± 4.07	174.46 ± 5.08	73.80 ± 9.85	24.24 ± 2.98

*Note*: A: Iento group; B: Moderato group; C: Allegretto group; D: Control group.

### 3.2 Analysis of HR and HRV indicators before exercise

No significant differences in HR and HRV were observed between the four groups (*p* > .05) (see Table 4).

**TABLE 4:** Analysis of HR and HRV indicators before exercise ($\bar{x}\pm s$).

Index	A (44)	B (44)	C (41)	D (44)	*F*	*p*
HR (beats/min)	75.86 ± 11.42	75.77 ± 11.36	77.22 ± 12.01	78.59 ± 12.07	0.566	.638
SDNN (ms)	46.45 ± 22.56	52.61 ± 17.89	47.92 ± 21.45	52.64 ± 19.41	1.071	.363
LF (ms^2^)	6.81 ± 1.10	6.52 ± 0.98	6.31 ± 0.83	6.72 ± 0.88	2.318	.077
HF (ms^2^)	6.02 ± 1.06	5.89 ± 0.91	5.55 ± 0.94	5.64 ± 0.96	2.153	.095
LF_norm_ (n.u.)	52.90 ± 3.61	52.71 ± 3.51	53.42 ± 3.37	54.54 ± 3.52	2.411	.069
HF_norm_ (n.u.)	47.10 ± 3.61	47.33 ± 3.51	46.58 ± 3.37	45.46 ± 3.52	2.483	.063
LF/HF	1.15 ± 0.17	1.12 ± 0.15	1.15 ± 0.15	1.21 ± 0.16	2.555	.057

*Note*: Group A, *lento* (slow); group B, *moderato* (moderate); group C, *allegretto* (fast); group D, control group.

### 3.3 Analysis of the differences in the outcome measures of the power bicycle test during high‐intensity exercise among the four groups

The absolute and relative values of average power, absolute and relative values of maximum power, fatigue index, maximum power arrival time, and total power among the four groups were not among the statistical significance (*p* > .05) (Table 5).

**TABLE 5:** Analysis of differences in the outcome measures of the power bicycle test during high‐intensity exercise among the four groups ($\bar{x}\pm s$).

Wingate	A (44)	B (44)	C (41)	D (44)	*F*	*p*
MP						
watt	605.65 ± 152.47	594.09 ± 157.78	604.91 ± 139.28	650.68 ± 84.87	1.484	.221
watt/kg	6.75 ± 1.39	6.74 ± 1.39	6.73 ± 1.34	6.36 ± 0.93	0.943	.421
PP						
watt	970.48 ± 260.21	944.75 ± 315.38	953.46 ± 242.84	1034.64 ± 180.81	1.113	.345
watt/kg	12.88 ± 2.80	13.13 ± 3.06	12.79 ± 2.54	14.09 ± 2.18	2.173	.093
FI	20.08 ± 7.43	21.00 ± 10.08	18.83 ± 6.55	17.91 ± 3.50	1.531	.208
PPI						
sec	4.23 ± 0.25	4.23 ± 0.30	4.16 ± 0.31	4.32 ± 0.31	2.197	.090
TW						
watt	18,349.36 ± 4384.33	17,822.59 ± 4733.41	18,147.22 ± 4178.26	19,520.45 ± 2546.23	1.464	.226

*Note*: Group A, *lento* (slow); group B, *moderato* (moderate); group C, *allegretto* (fast); group D, control group.

Abbreviations: FI, fatigue index; MP, mean power; PP, peak power; PPI, maximum power arrival time; TW, total power.

### 3.4 Differences in the changes in the HRR and HRV indexes over time with the music intervention during the recovery period after high‐intensity exercise

**FIGURE 1:**
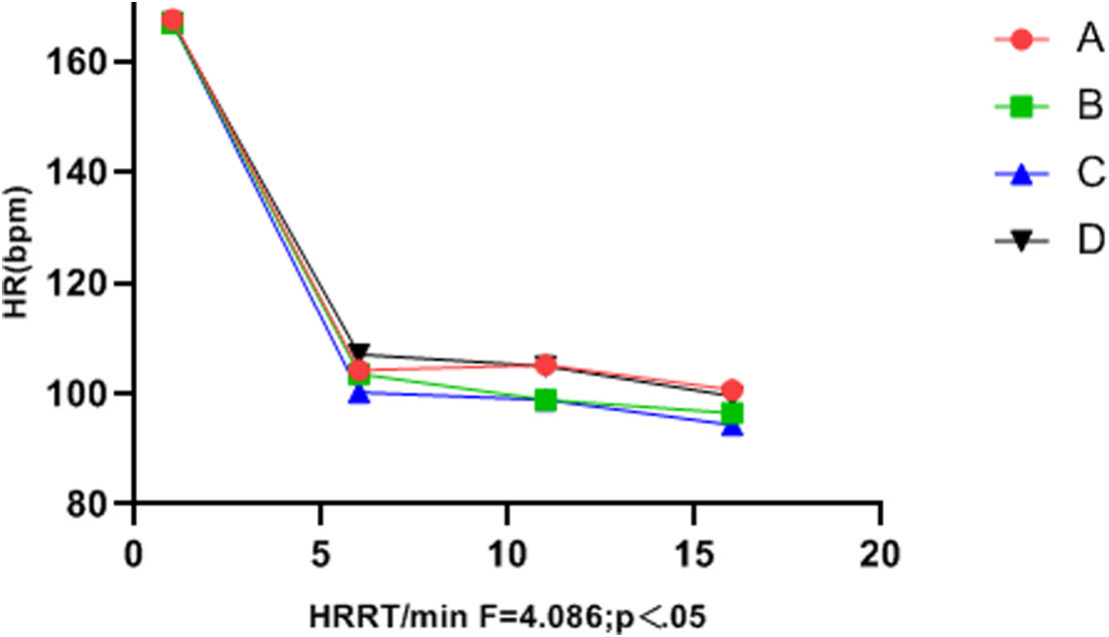
Illustration of experimental results. Group A, *lento* (slow); group B, *moderato* (moderate); group C, *allegretto* (fast); group D, control group.

**FIGURE 2:**
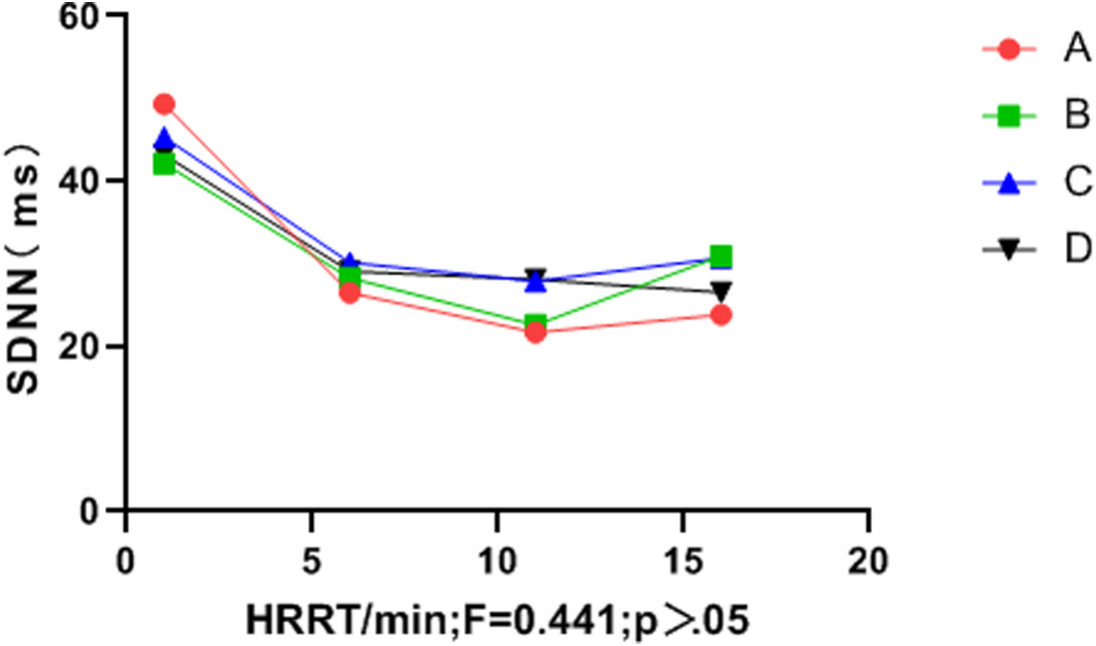
Time‐domain variation index SDNN in the recovery period.

Heart rate differed significantly between the groups (*p* < .05), and the HRR of group C was superior to that of groups A and B. In addition, groups B and C were superior to group D (control). Although SDNN did not differ significantly between groups (*p* > .05), the final SDNN values in groups B and C were higher than those in groups A and D (Table 6, Figures 1 and 2).

**TABLE 6:** Analysis of the HRR time domain variation index during the recovery period ($\bar{x}\pm s$).

Index	Recovery period (min)	A (44)	B (44)	C (41)	D (44)	Grouping	*F*	*p*	Post‐hoc
HR (N/min)	0–1	167.55 ± 3.32	166.91 ± 3.52	167.08 ± 3.57	167.80 ± 3.46	Frequency	49.12	.000	D > B, C; A > C
	5–6	104.16 ± 10.66	103.50 ± 11.79	100.24 ± 12.56	107.00 ± 10.12	Rhythm	4.086	.008	
	10–11	105.16 ± 9.50	98.86 ± 12.99	98.88 ± 13.20	104.91 ± 10.73	Frequency	1.716	.166	
	15–16	100.66 ± 9.65	96.45 ± 13.96	94.29 ± 12.67	99.50 ± 11.86	Rhythm			
SDNN (ms)	0–1	49.33 ± 23.78	42.12 ± 20.39	45.31 ± 23.65	43.16 ± 25.80	Frequency	32.09	.000	NS
	5–6	26.45 ± 23.78	28.27 ± 18.07	30.10 ± 27.56	29.05 ± 29.82	Rhythm	0.441	.724	
	10–11	21.69 ± 19.39	22.58 ± 15.34	27.85 ± 21.93	28.05 ± 13.07	Rhythm	0.942	.488	
	15–16	23.83 ± 22.96	30.93 ± 23.08	30.68 ± 22.02	26.50 ± 24.05				

### 3.5 Analysis of HRV frequency domain indicators in the recovery period

**FIGURE 3:**
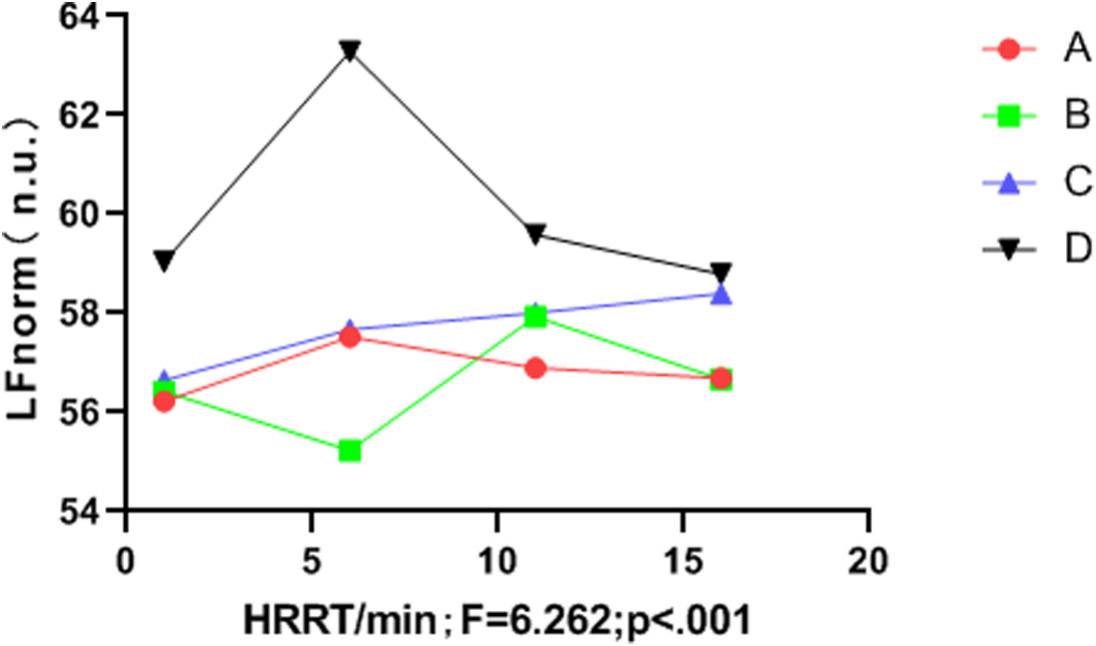
Frequency domain indices LF_norm_ in the recovery period.

**FIGURE 4:**
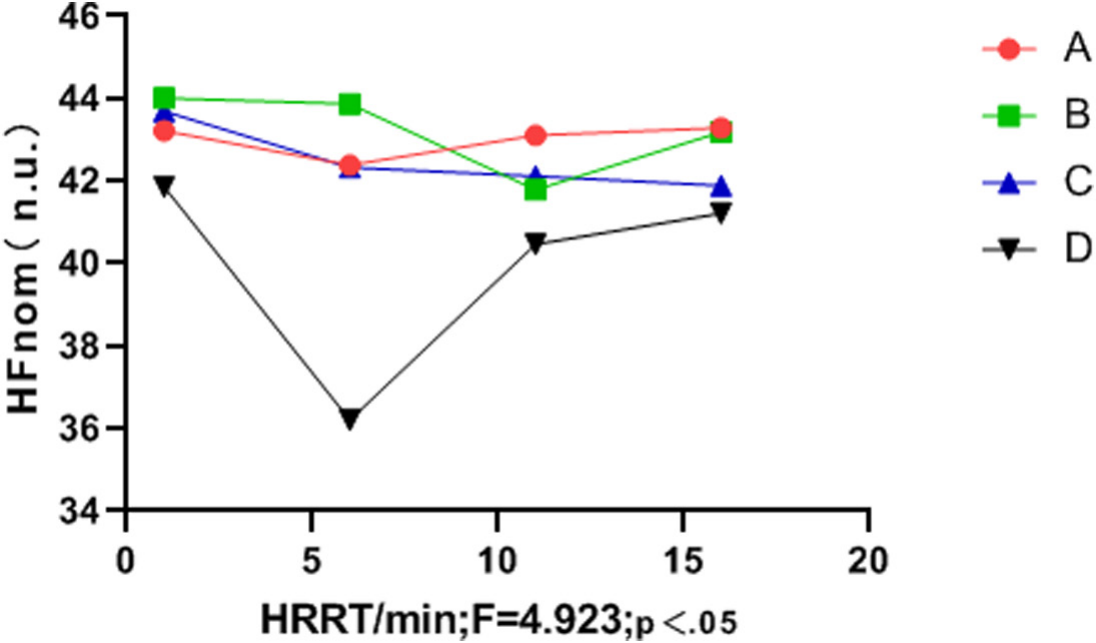
Frequency domain indices HF_norm_ in the recovery period.

**FIGURE 5:**
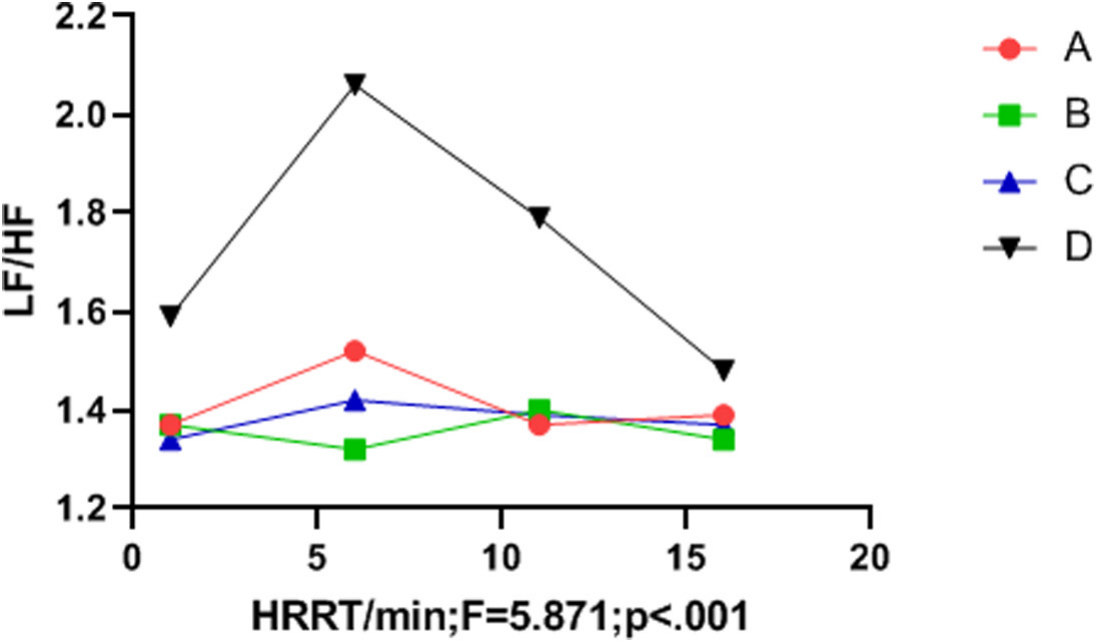
Frequency domain indices LF/HF_norm_ in the recovery period.

The frequency domain variation indicators LF_norm_, HF_norm_, and LF/HF differed significantly between groups (*p* < .05). The music intervention groups (A, B, and C) had significantly better LF/HF than the control group (D), and the LF/HF value of group C was more stable than that of groups A, B, and D (see Table 7, Figures 3, 4, 5).

**TABLE 7:** Analysis of HRV frequency domain indicators during the recovery period ($\bar{x}\pm s$).

Index	Recovery period (min)	A (44)	B (44)	C (41)	D (44)	Grouping	*F*	*p*	Post‐hoc
LF (ms^2^)	0–1	6.28 ± 1.49	6.18 ± 1.44	6.13 ± 1.62	5.63 ± 1.75	Frequency	34.61	.000	B, C > D
	5–6	4.83 ± 1.72	5.02 ± 1.67	4.80 ± 1.50	4.50 ± 1.67	Rhythm	2.768	.043	
	10–11	4.57 ± 1.28	4.65 ± 1.62	4.86 ± 1.54	4.13 ± 1.67	Frequency*	0.45	.907	
	15–16	4.90 ± 1.36	5.35 ± 1.51	5.19 ± 1.47	4.93 ± 1.56	Rhythm			
HF (ms^2^)	0–1	4.83 ± 1.36	4.81 ± 1.48	4.82 ± 1.55	3.89 ± 1.71	Frequency	21.012	.000	A, B, C > D
	5–6	3.66 ± 1.78	4.13 ± 1.56	3.61 ± 1.42	3.08 ± 1.93	Rhythm	7.151	.000	
	10–11	3.54 ± 1.27	3.68 ± 1.48	3.73 ± 1.54	2.92 ± 1.68	Rhythm*	0.632	.770	
	15–16	3.70 ± 1.19	4.15 ± 1.41	4.07 ± 1.58	3.55 ± 1.32	Frequency			
LF_norm_ (n.u.)	0–1	56.22 ± 4.48	56.42 ± 4.04	56.65 ± 5.19	59.04 ± 4.50	Frequency	1.676	.171	D > A, B, C
	5–6	57.52 ± 7.50	55.23 ± 8.25	57.67 ± 6.19	63.28 ± 10.63	Rhythm	6.262	.000	
	10–11	56.90 ± 6.38	57.93 ± 7.87	58.00 ± 5.08	59.58 ± 9.75	Frequency*	2.113	.027	
	15–16	56.69 ± 7.01	56.66 ± 5.13	58.40 ± 5.53	58.79 ± 5.85	Rhythm			
HFnom (n.u.)	0–1	43.21 ± 4.09	44.00 ± 4.23	43.68 ± 5.15	41.86 ± 4.90	Frequency	3.15	.025	A, B, C > D
	5–6	42.38 ± 7.41	43.87 ± 8.90	42.33 ± 6.19	36.20 ± 13.04	Rhythm	4.923	.003	
	10–11	43.10 ± 6.38	41.78 ± 7.88	42.12 ± 5.10	40.46 ± 9.73	Rhythm*	2.075	.030	
	15–16	43.28 ± 6.99	43.18 ± 5.11	41.88 ± 5.44	41.21 ± 5.85	Frequency			
LF/HF	0–1	1.37 ± 0.33	1.37 ± 0.34	1.34 ± 0.37	1.59 ± 0.42	Frequency	3.623	.013	A, B, C > D
	5–6	1.52 ± 0.69	1.32 ± 0.46	1.42 ± 0.40	2.06 ± 1.74	Rhythm	5.871	.001	
	10–11	1.37 ± 0.36	1.40 ± 0.57	1.39 ± 0.33	1.79 ± 1.36	Frequency*	2.089	.029	
	15–16	1.39 ± 0.41	1.34 ± 0.29	1.37 ± 0.39	1.48 ± 0.43				

*Note*: Group A, *lento* (slow); group B, *moderato* (moderate); group C, *allegretto* (fast); D, control group.

## 4 DISCUSSION

Multiple studies have revealed a significant correlation between vigorous and high‐intensity exercise and the risk of SCD and AMI (Balnave et al., 2013; Cole et al., 1999; He et al., 2019). Heart rate recovery (HRR) and heart rate variability (HRV) are also key predictors of cardiovascular disease and sudden malignant cardiac events in healthy populations (Kannankeril et al., 2004). Previous reports have linked HR to heart failure (Wang & Zheng, 2006), coronary heart disease (Bigger et al., 1988), sudden death (Imai et al., 1994), atrial fibrillation (Stefan & Jäncke, 2015), and other cardiovascular diseases. HR is closely associated with imbalances in HRV and autonomic nervous system regulation (Iwanaga et al., 2005). A shorter HRR time after exercise is correlated with a lower incidence of cardiovascular disease, whereas delayed parasympathetic activation after exercise may increase the risk of sudden cardiac death (Orini et al., 2010). In the present study, the four groups showed no significant differences in the HRR or HRV indices before vigorous exercise (*p* > .05). Similarly, during vigorous exercise, no differences were observed in the anaerobic power cycling indices between groups (*p* > .05). However, during the recovery period, listening to music significantly influenced HRR, LF, HF, LF_norm_, HF_norm_ and the LF/HF ratio (*p* < .05). In terms of HRR, Group C outperformed Groups B and A, and Groups B and C outperformed Group D (control). Although the standard deviation of SDNN intervals did not differ between music intervention groups (*p* > .05), Figure 2 indicates that the final SDNN values of Groups B and C were higher than those of Group A and Group D (control). Additionally, the music intervention groups (A, B, and C) demonstrated significantly more improvement in the LF_norm_ and HF_norm_ indices than Group D. Figure 5 demonstrates that the music intervention groups (A, B, and C) outperformed Group D in the LF/HF ratio, and Group C showed a more stable LF/HF value than Groups A, B, and D.

During and after physical exercise, HR increases sharply, parasympathetic nervous system activity decreases, and sympathetic nervous system activity increases; this elevated sympathetic activity during exercise increases the likelihood of sudden cardiac events (Perkins et al., 2015). However, during the recovery period, HR is negatively correlated with the risk of sudden cardiac events (Hernesniemi et al., 2020). An increase in HRV, specifically the SDNN intervals, indicates increased parasympathetic nervous system activity, raising the threshold for ventricular fibrillation and thus lowering the probability of ventricular fibrillation. Conversely, a decrease in SDNN implies damage to the cardiac autonomic nervous system, which may result in unstable myocardial electrical activity and a lower threshold for ventricular fibrillation, thereby increasing the risk of malignant arrhythmias and sudden cardiac death (Hernesniemi et al., 2020). Most arrhythmia‐related sudden cardiac deaths are caused by ventricular tachyarrhythmias. Lower HRV signifies decreased parasympathetic nervous system activity and increased sympathetic nervous system activity, leading to a lower ventricular fibrillation threshold. In addition, increased LF, decreased HF, and an elevated LF/HF ratio can trigger cardiac events (Pezawas et al., 2013; Stefanović & Milojković, 2018). Therefore, the reactivation of the parasympathetic nervous system and regulation of the LF/HF ratio after exercise can rapidly and effectively protect the heart.

Therefore, this study used different music speeds as interventions after intense and high‐intensity exercise. Group C (allegretto) showed superior HRR during the recovery period compared with Group B (moderato) and Group A (lento) (*p* < .05). Additionally, HRR time was lower in both Groups B and C than in Group D (control). The final SDNN interval values in Groups B and C were higher than those in Groups A and D, although the difference was not statistically significant (*p* > .05). The change curves of the LF_norm_ and HF_norm_ indices (*p* < .05) indicated that the music intervention groups (A, B, and C) significantly outperformed Group D in these indices. Moreover, the LF/HF ratio in Groups B and C was more stable than that in Groups A and D. These results indicate that listening to allegretto music (Group C) after intense and high‐intensity exercise had the most significant impact on HRR and the greatest regulatory effect on HRV. Thus, listening to music at this tempo could potentially aid in preventing and reducing the risk of arrhythmias and sudden cardiac events after high‐intensity exercise.

## 5 CONCLUSIONS

After rigorous activity, listening to allegretto transcriptions improved HRR and restored HRV equilibrium, which is critical for preventing and minimizing arrhythmia and SCD.

Music is an effective tool for regulating mood and autonomic function; it also has potential as a low‐cost, safe intervention and therapeutic adjunct, The melody, tempo, volume, and other variables may have varying impacts on HRR and HRV. Consequently, finely controlling variables and conducting further research are required to accurately determine which music tempo is most effective for recovery after high‐intensity exercise; in addition, the volume and timbre of the music the length of the recovery period and intervention, and other factors may have varying effects on the autonomic nervous system. As a result, researchers in this field should continue to investigate the use of music to prevent and reduce the risk of cardiovascular incidents.

## AUTHOR CONTRIBUTIONS

Niu Mingyang and Wang Jiameng designed the experiment, Niu Mingyang and Zhao Ruixue collected the data, Wang Jiameng conducted the data analysis, Niu Mingyang and Zhao Ruixue provided experimental guidance, and Wang Jiameng and Niu Mingyang completed the manuscript writing and the revision of important content.

## FUNDING INFORMATION

This article was supported by the 2022 Hainan Province Philosophy and Social Science Planning Project (HNSK(QN)22‐84) and the 2021 Project supported by the Education Department of Hainan Province (project number: Hnky2021‐41).

## CONFLICT OF INTEREST STATEMENT

The authors declare no conflicts of interest.

## ETHICS STATEMENT

The experiment was performed after an informed consent form was signed by each participant and confirming that none of the students had cardiovascular or other lethal diseases. The study was conducted in accordance with the Declaration of Helsinki and approved by the Bioethics Commission of Hainan Medical University (HYLL‐2021‐369).

## Data Availability

The raw data supporting the conclusions of this article will be made available by the authors upon reasonable request.
